# Limitations of homologous recombination status testing and poly (ADP-ribose) polymerase inhibitor treatment in the current management of ovarian cancer

**DOI:** 10.3389/fonc.2024.1435029

**Published:** 2024-07-22

**Authors:** Qianying Zhao, Liping Bai, Yu Tan, Mingrong Qie

**Affiliations:** ^1^ Department of Medical Genetics/Prenatal Diagnostic Center, West China Second University Hospital, Sichuan University, Chengdu, China; ^2^ Key Laboratory of Birth Defects and Related Diseases of Women and Children (Sichuan University), Ministry of Education, Chengdu, China; ^3^ Department of Obstetrics and Gynecology, West China Second University Hospital, Sichuan University, Chengdu, China

**Keywords:** homologous recombination (HR), PARP inhibitor (PARPi), ovarian cancer (OC), future prospect, limitations

## Abstract

Homologous recombination (HR) is a highly conserved DNA repair system, in which aberrations can lead to the accumulation of DNA damage and genomic scars known as homologous recombination deficiency (HRD). The identification of mutations in key genes (i.e., *BRCA1*, and *BRCA2* (BRCA)) and the quantification of large-scale structural variants (e.g., loss of heterozygosity) are indicators of the HRD phenotype. HRD is a stable biomarker and remains unchanged during recurrence, but fails to reveal the molecular profile of tumor progression. Moreover, interpretation of the current HRD score lacks comprehensiveness, especially for the HR-proficient group. Poly (ADP-ribose) polymerase (PARP) enzymes play an important role in the repair of DNA single-strand breaks, the blockage of which using PARP inhibitors (PARPi) can generate synthetic lethality in cancer cells with HRD. Although numerous studies have demonstrated that the benefit of PARPi is substantial in ovarian cancer (OC) patients, the efficacy is limited by the development of resistance, and seems to be irrespective of HR and/or BRCA mutation status. Moreover, in addition to improving progression-free survival, long-term benefit as overall survival brought by PARPi for advanced, recurrent and refractory OC patients remains unclear. Therefore, further investigations are needed to uncover the role of HR genes beyond BRCA and their interactions with other oncogenic pathways, to determine the value of HRD in the recurrent setting, and to identify alternative strategies for the precise management of advanced, refractory OC patients.

## 1 Introduction

High-grade serous carcinoma of ovary, fallopian tube or peritoneum is one of the most common gynecological malignancies. High−grade serous ovarian cancer (HGSOC) represents 70% of all ovarian cancers (OC). Although most patients achieve clinical remission following initial treatment by standard cytoreductive surgery and platinum-based chemotherapy, approximately 70% of patients still suffer recurrence, and the average 5-year survival rate is approximately 30% (1, 2). Even with considerable investigations, effective screening methods for early diagnosis, as well as precise stratification modality of individualized clinical management are lacking.

**Figure 1:**
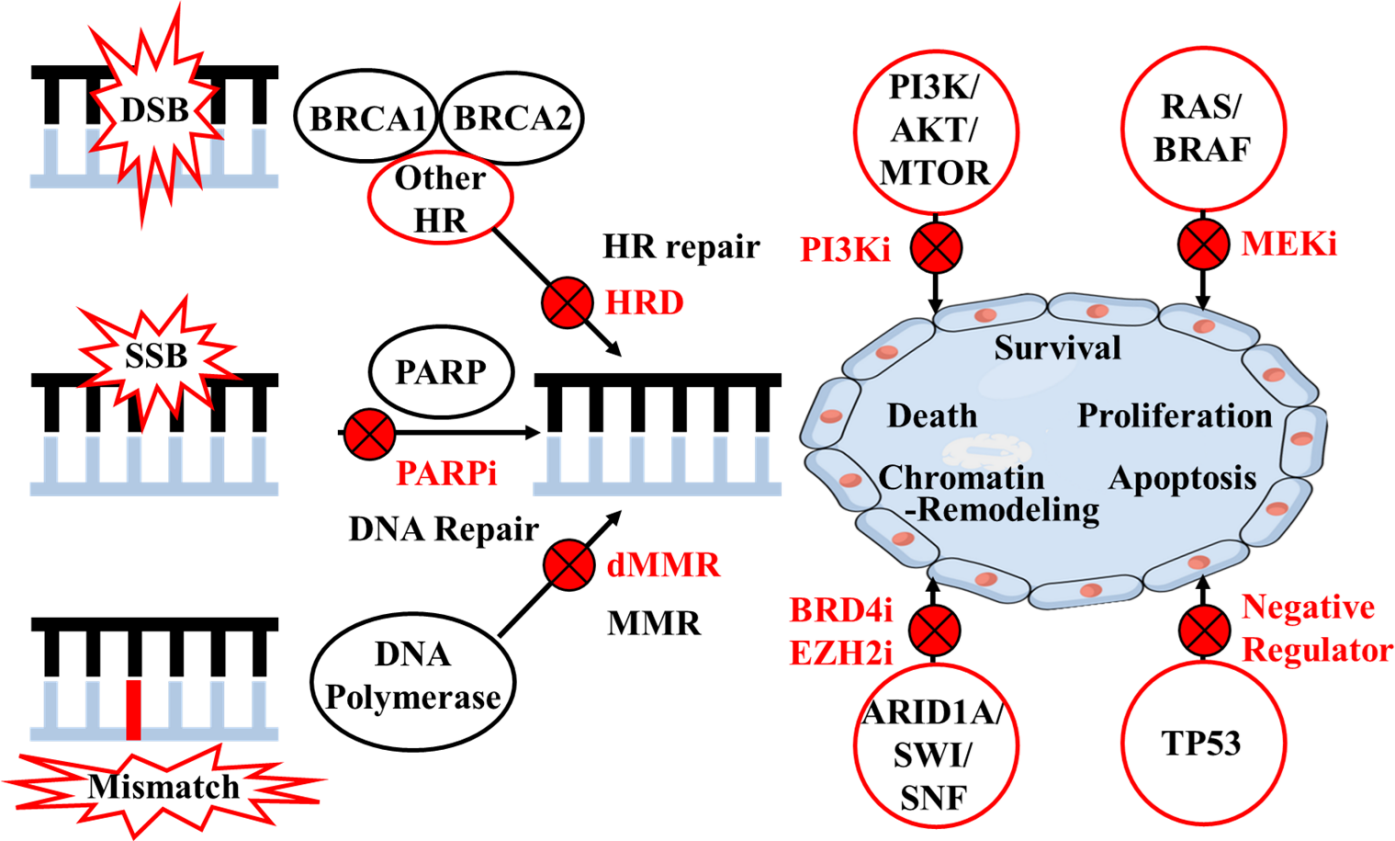
Mechanisms of target therapies according to homologous recombination and other pathways. DSB, double-strand DNA break; SSB, single-strand DNA break; HR, homologous recombination; HRD, homologous recombination deficiency; PARP, poly (ADP-ribose) polymerase; i, inhibitor; MMR, mismatch repair; dMMR, mismatch repair deficiency.

Homologous recombination (HR) system is responsible for double-strand DNA breaks (DSB) repair with high-fidelity. HGSOC with homologous recombination deficiency (HRD) exhibits distinct clinical features, including a superior response to platinum-based chemotherapies and sensitivity to poly (ADP-ribose) polymerase (PARP) inhibitors (PARPi) (3, 4). PARP enzymes can repair single-strand DNA breaks; therefore, PARPi can achieve “synthetic lethality” when affecting tumor cells with HRD as shown in **Figure 1** (5). Numerous clinical studies have demonstrated that certain OC patients, either newly diagnosed or with recurrent disease, may benefit from PARPi as maintenance or recurrence treatment after primary platinum-based treatment (6). The introduction of PARPi has transformed the management of HGSOC in both first-line treatment and relapsed setting (7–12). Correspondingly, molecular analysis is recommended by guidelines (e.g., National Comprehensive Cancer Network, NCCN) to include germline or somatic *BRCA1* and *BRCA2* (BRCA) gene mutations, loss of heterozygosity (LOH) or HR status in OC tissues (6, 13).

However, the stratification of OC patients based on BRCA mutations and LOH testing still lacks efficiency, especially for recurrent, refractory, and drug-resistant cases. Despite the improvement of progression-free survival (PFS) associated with PARPi, tailored management and benefit of overall survival (OS) for OC patients are still challenging. Herein, we conclude and address the limitations of the current molecular testing and PARPi treatment for OC patients in terms of both bio-pathological mechanisms and clinical circumstances.

## 2 Limitations of HR testing and PARPi in OC patients

### 2.1 Complexity of HR status and related testing

HRD, also known as “genomic scars”, refers to cellular-level impairment of the HR repair system, leading to quantifiable and stable genomic changes. Currently, HR status is typically evaluated by BRCA mutations, LOH, telomeric allelic imbalance (TAI), and large-scale state transition (LST), with the genomic instability score (GIS) subsequently calculated. Mutations in BRCA genes are mainly identified through next-generation sequencing (NGS) and multiplex ligation-dependent probe amplification (MPLA), which is used for the identification of large rearrangement variants. HRD is a common molecular marker of tumor cells, and is frequently detected in metastatic OC tissues as HR gene mutations (14). The Cancer Genome Atlas (TCGA) project revealed that 22% of HGSOC patients had germline and/or somatic mutations in BRCA genes, and HR was defective in approximately half of the tumors (15). At present, HRD has rapidly developed as a therapeutic predictor for PARPi, especially for OC patients.

However, HRD is a stable genomic marker for which the evaluation is not affected by the tumor sampling site. Research conducted in 50 paired HGSOC samples suggested that HRD was maintained between the primary and recurrent lesions, regardless of other genomic changes that occurred during recurrence (16). This study expands the applicable population of OC patients to receive PARPi treatment based on HR status. However, HR status may not accurately represent the molecular features of progressive or recurrent tumor subclones, as genomic scars can be detected with the same-level HRD score. Moreover, studies in ovarian and prostate cancer have revealed multiple-gene (e.g., *BRCA1* and *BRCA2*) reversion mutations merely identified in progressive tumor tissues, which lead to the restoration of DNA repair function and acquired drug resistance. However, traces of genomic scars caused by HRD did not disappear in such progressive lesions, which could cause inappropriate stratification of patients depending solely on HR status (17, 18). Extensive characterization of the molecular events contributing to drug resistance or disease relapse has largely been hindered, partially due to the failure to acquire sequential samples from the same cancer patient throughout the course of their disease. In particular, collecting OC tissue samples during relapse for molecular profiling is challenging, since numerous progressive lesions are unresectable. For such recurrent cases, circulating tumor DNA would be a suitable sample source.

In addition, the negative results of the current HR test have limited predictive value and cannot fully explain the complexity of the HRD phenotype in each subgroup (19, 20). In addition to the wide use of the positive cutoff HRD score of ≥42, a recent study suggested that patients with HRD scores <33 were less likely to benefit from platinum-based chemotherapy, and had a worse PFS than patients in the HRD-negative or HRD ≥33 BRCA- mutated (BRCAm) group (21, 22). However, precise subgrouping of HR- proficient patients using appropriate HRD cutoff score warrants further investigations in cohorts with large sample sizes. Genomic scar analysis of somatic copy number variant based on single nucleotide polymorphisms (SNPs) is currently the most widely applied technique for HR testing. The interpretation of HR status should be handled cautiously according to the cutoff acquired from different scoring systems for specific PARPi and for corresponding pathological cancer types in distinct ethnic groups.

In summary, both positive and negative results of the current HR test have limitations for clinical prediction. Detection involving more aspects (e.g., promoter methylation of BRCA genes and mutations of HR genes beyond BRCA) can more comprehensively reveal the nature of HR system and identify more accurate beneficiaries of PARPi, especially from the BRCA wild-type (BRCAwt) and/or HR-proficient patients.

### 2.2 Therapeutic effect of PARPi irrespective of HR or BRCA mutation status

A number of phase III randomized controlled clinical trials (e.g., SOLO-1, PRIMA) have completed and verified the remarkable efficacy of PARPi as first-line maintenance therapy for certain OC patients, with delayed relapse and prolonged PFS (9, 10). Specifically, subgroup analysis demonstrated that BRCA mutation carriers and the HRD group benefited more than those in the HR-proficient group. It is likely that synthetic lethality occurs for patients with HRD (germline or somatic) when exposed to PARPi. According to TCGA, approximately half HGSOC patients were thought to have a deficiency in HR system, expanding the availability of maintenance therapy by PARPi for a significant number of women with advanced OC (15).

However, a portion of these studies revealed that the overall benefit of PARPi was substantial in patients irrespective of HR or BRCA mutation status (**Table 1**). Study 19 (a randomized phase II trial in platinum-sensitive, relapsed HGSOC patients who had received two or more platinum-based regimens and who had a partial or complete response to their most recent platinum-based regimen) demonstrated that a PFS advantage was also seen for BRCAwt patients. An apparent OS improvement was observed with olaparib compared with placebo (hazard ratio 0.73, P =0.02138), irrespective of BRCA mutation status (23). The phase III OPINION trial investigated olaparib maintenance monotherapy in patients without a germline BRCA mutation (gBRCAm) who had platinum-sensitive relapsed OC and had received ≥2 previous lines of platinum-based chemotherapy. It revealed a clinical benefit from maintenance olaparib in patients without a gBRCAm, and across all subgroups compared with placebo control individuals (24). In addition, the L-MOCA study indicated that regardless of BRCA status, olaparib maintenance therapy significantly benefited Asian patients with platinum-sensitive recurrent disease in terms of PFS (25). Similarly, a phase III randomized controlled study in China (NORA) also indicated that both gBRCAm and non-gBRCAm carriers with platinum-sensitive recurrent OC benefited from niraparib treatment with longer PFS (26). Notably, the result of the OReO trial recruiting patients who heavily pre-treated with receiving ≥3 prior lines of any chemotherapy, demonstrated the non-BRCA mutation cohort had better PFS than BRCA mutation cohort with maintenance olaparib rechallenge (27). Another nonrandomized study of olaparib monotherapy in patients with OC reported response rates of 41% and 24% in the gBRCAm and nonmutated BRCA patient populations, respectively (31).

**Table 1:** Clinical trials in OC relevant to PARPi and HR status.

Trial	Phase	Region	OC Population	Patient no.	PARPi	PFS regardless of HR status	OS benefit by PARPi*
SOLO-1 (10)	III	Global	Newly diagnosed advanced	391	Olaparib	NA	No
PRIMA (9)	III	Global	Newly diagnosed advanced	733	Niraparib	Yes	No
Study 19 (23)	II	Global	Platinum-sensitive recurrent	265	Olaparib	Yes	No
OPINION (24)	III	Global	Platinum-sensitive relapsed	279	Olaparib	Yes	NA
L-MOCA (25)	III	Asian	Platinum-sensitive relapsed	225	Olaparib	Yes	NA
NORA (26)	III	China	Platinum-sensitive recurrent	265	Niraparib	Yes	NA
OReO (27)	III	Global	Platinum-sensitive relapsed	220	Olaparib	Yes	NA
SOLO-2 (11, 28)	III	Global	Platinum-sensitive relapsed	295	Olaparib	NA	No
SOLO-3 (29)	III	Global	Platinum-sensitive relapsed	266	Olaparib	NA	No
NOVA (30)	III	Global	Platinum-sensitive recurrent	553	Niraparib	Yes	No

OC, ovarian cancer; no., number; PARPi, poly (ADP-ribose) polymerase inhibitor; PFS, progression-free survival; HR, homologous recombination; NA, not applicable; OS, overall survival.

*: if OS has been improved significantly by PARPi compared with placebo, it is indicated as “YES”, otherwise as “No”.

Overall, HR status and/or BRCA mutation testing may lack adequate efficacy as inclusion criteria for PARPi treatment, especially in recurrent or refractory OC patients. Since the HR-proficient group could also benefit, the use of PARPi in the candidate OC population could be broadened irrespective of HR or BRCA mutation status. In addition, it is a challenge to further distinguish which patients with BRCAwt or HR proficiency are most likely to benefit from PARPi.

### 2.3 Indeterminate benefit of PARPi on long-term survival

Although it has been verified by abundant studies that PFS has been remarkably prolonged, the results from several large-scale multicenter clinical studies have suggested that improvements in OS have not yet achieved statistical significance for advanced OC patients treated with PARPi therapy (**Table 1**).

Study 19 revealed that an apparent OS advantage was observed with olaparib vs. placebo (hazard ratio 0.73, P =0.02138); however, this difference did not meet the preset threshold defined for statistical significance (P <0.0095). There was little difference in the median endpoint estimates (29.8 months for olaparib vs. 27.8 months for placebo), which was unsatisfactory for advanced OC patients (23). SOLO-2 (a double-blind, randomized, placebo-controlled, phase III trial was done across 123 medical centers in 16 countries) demonstrated that the median OS was 51.7 months and 38.8 months with olaparib and placebo, respectively. Although olaparib provided a median 12.9-month benefit of OS in patients with platinum-sensitive relapsed OC, the difference was not statistically significant (hazard ratio 0.74; p =0.054) (28). The phase III SOLO-3 study showed a significant objective response and improvement in PFS in patients with gBRCAm platinum-resistant or partially platinum-sensitive relapsed OC who were treated with olaparib capsules. Long-term endpoint analysis was performed at approximately 60% data maturity for OS. However, OS was similar in the olaparib and the TPC (single-agent non-platinum chemotherapy of physician’s choice) group (29). NOVA trial was a multicenter, double-blind, phase III, randomized controlled trial among patients with platinum-sensitive recurrent OC. Maintenance therapy with niraparib prolonged PFS regardless of the presence or absence of gBRCAm or HRD. Similarly, during the study follow-up period, 60 of 372 patients (16.1%) in the niraparib group and 35 of 181 (19.3%) in the placebo group died, with more complete OS data available (30).

Overall, the long-term PARPi benefit (i.e., OS) for advanced OC patients is indeterminate and far from satisfactory. These findings are still arguable lacking adequate long-term follow-up results. There is also an urgent need for more treatment alternatives for OC patients without specific molecular profiles, but with poor prognosis (15).

## 3 Future prospects

Due to the aforementioned limitations, biomarkers beyond BRCA mutations and/or HR status are under continuous investigation for a significant proportion of advanced, relapsed or refractory OC patients. It has long been recommended by NCCN guidelines to include BRCA genes, HR status, microsatellite instability (MSI), tumor mutation burden (TMB) and NTRK evaluation when encountering recurrent OC patients. If necessary, further molecular testing should be conducted to explore potential treatments and improve patients’ prognosis (6).

### 3.1 HR genes beyond BRCA

HR system is a complex signaling pathway involving multiple steps and factors, and protein-encoding genes other than *BRCA1* and *BRCA2* include *ATM*, *RAD51*, *PALB2*, *MRE11*, *RAD50*, *NBN*, *CDK12*, and *FA* (16). A retrospective analysis of the Study 19 cohort also suggested that the HGSOC subgroup with other HR gene (e.g. *BRIP1*, *CDK12*, *RAD54L*, *RAD51B*) mutations derived similar benefits from PARPi as did the BRCA mutation group (21). ARIEL3 study in OC showed that rucaparib-arm patients with *RAD51C* or *RAD51D* alterations had very high frequency of exceptional benefit from PARPi (32). *In vitro* studies also suggested that defects in other HR genes including *ATM*, *CHEK1/2*, *NBN*, *PALB2*, *MRE11A* and *RAD50* could also reflect sensitivity to PARPi (1, 33–35). Research has shown germline and/or somatic mutations in HR genes exist in both serous and nonserous OC, including clear cell, endometrioid, and carcinosarcoma, which might also benefit from PARPi (4). Additionally, research findings in other malignancies could shed light upon OC studies: the PROfound study demonstrated that olaparib could reduce disease progression or death by 66% in patients with metastatic castration-resistant prostate cancer (mCRPC) carrying deleterious mutations in *BRCA1*, *BRCA2* and *ATM*. Other pathogenic mutation carriers in other HR genes such as *BARD1*, *BRIP1*, *CDK12*, *CHEK1/2*, *FANCL*, *PALB2*, *RAD51B/C/D*, and *RAD54L* also had improved PFS (36). Therefore, the role of other HR genes and their interactions with BRCA genes should continue to be the subject of further OC investigations, and inhibition of such DNA repair pathways would expand the use of PARPi (**Figure 1**).

### 3.2 Other pathways beyond HR system

The identification of new therapeutic approaches targeting other pathways is also a critical direction of investigations relevant to OC. The TCGA database revealed that recurrent somatic mutations occurred in genes including *NF1*, *RB1*, and *CDK12* (15). In addition to HR system, commonly deregulated pathways include RB, RAS/PI3K, FOXM1, and NOTCH pathways, and genes involved more broadly in the DNA damage repair system (e.g., *MSH2*, *MUTYH*) provide opportunities for therapeutic attack (21). The phosphatidylinositol-3 kinase (PI3K) pathway is a crucial intracellular signaling pathway that is mutated or amplified in a wide variety of cancers including breast, gastric, ovarian, colorectal, prostate, and endometrial cancers and glioblastoma (37). The research sequencing 410 genes of 82 ovarian carcinomas showed that gain of *PIK3CA* was characteristic of HGSOC, with H1047R/L being the mutation hotspot (38). A multicenter phase I study showed that 12% RAS/BRAF-mutant advanced OC patients who achieved partial response were treated with binimetinib (MEK inhibitor) in combination with buparlisib (PI3K inhibitor) (39). However, the PI3K/AKT/MTOR pathway is also complex, and currently PI3K and AKT inhibition may be most promising in clear cell and endometrioid carcinomas of ovary (38). *ARID1A* is a member of the SWI/SNF chromatin remodeling complex that plays a role in various cellular function by altering chromatin structure (40). *In vitro* studies have demonstrated that *ARID1A* deficiency could sensitize cancer cell lines to PARPi, BRD4 inhibitor and EZH2 inhibitor (41–43). *TP53* encodes a tumor suppressor protein and contains transcriptional activation, DNA binding and oligomerization domains. The protein responses to a variety of cellular stresses, regulates the expression of target genes, and induces cell cycle arrest, apoptosis, senescence, DNA repair or metabolic changes (44). Approximately 50%-96% of HGSOCs harbor a clonal somatic *TP53* mutation (15, 45). Combined tumor BRCA/*TP53* mutation testing may provide an advantage of rapid results in comparison to gBRCAm testing via oncogenetic counseling. And combined tumor BRCA/*TP53* testing could also validate the presence of somatic BRCA mutations in samples with a low cellularity (46). Nultlin 3a inhibiting MDM−2 (a negative regulator of p53 protein) and adenovirus−mediated *TP53* gene transfer system is currently analyzed in a clinical trial, which may restore p53 activity and provide potential therapeutic target (44, 47). Part of the pathways beyond HR system and potential therapy under investigation are shown in **Figure 1**.

### 3.3 Other biomarkers beyond target genes

In addition to gene mutations, other molecular markers should be evaluated comprehensively. MSI is the result of mismatch repair deficiency, which is commonly accompanied by high TMB, and involved in tumor development and progression (**Figure 1**). Numerous studies have suggested that MSI/TMB is related to the efficacy of immune checkpoint inhibitors (ICIs), such as programmed cell death-1 (PD-1) and programmed cell death-ligand 1 (PD-L1) antibodies. Acceptable therapies recommended by NCCN include dostarlimab-gxly for recurrent or advanced epithelial OC tumors, and pembrolizumab for solid tumors either MSI-high or mismatch repair-deficient (dMMR) or TMB-high (≥10 mutations/megabase) (6).

TCGA has also analyzed mRNA expression, miRNA expression, promoter methylation, and copy number variants in a large cohort of HGSOC samples. Elevated promoter methylation events involved 168 genes with reduced tumor expression, and it was notable that promoter hypermethylation of *AMT*, *CCL21* and *SPARCL1* were detected in the vast majority of tumors (15). In addition, a higher median HRD score was observed in BRCAm and BRCAwt tumors with *BRCA1* methylation (21). However, 75% of BRCAwt patients with *BRCA1*-methylated tumors, neither treated by olaparib or placebo, had disease progression. It may be because *BRCA1* methylation is unable to phenocopy *BRCA1* mutation in terms of olaparib sensitivity/ease of reversibility, which has also been reported for platinum sensitivity (4). Other study showed methylation of *BRCA1* was not associated with long-term olaparib response in OC patients, which warrants further evidences (48). Mass spectrometry-based proteogenomic characterization analysis of HGSOC showed that proteomic clusters had a clear correspondence to the mesenchymal, proliferative, immunoreactive, and differentiated subtypes defined by the TCGA transcriptome analysis. And specific protein acetylation associated with HRD suggested a potential means for stratifying patients for therapy (49).

## 4 Conclusions

While data from ceaseless OC studies are encouraging, challenges remain. OC patients with tumors harboring deleterious mutations in HR genes beyond BRCA may constitute a small, molecularly identifiable and clinically relevant population who benefit from PARPi treatment as patients with BRCA mutations or HRD. On the other hand, seeking biomarkers that aim to exclude those patients least likely to benefit may be alternative subject. Due to different bio-pathological roles and low mutation prevalence of each gene, integrated analysis involving the genome, transcriptome and proteome dimensions may provide a panoramic view of the molecular components and underlying mechanisms associated with OC. Subsequently, more precise stratification of OC patients could be achieved, leading to tailored management with favorable prognosis. Moreover, artificial intelligence, which is proficient in massive data processing using noncustomary algorithm can aid in uncovering the complicated nature of malignancies.

## Author contributions

QZ: Conceptualization, Resources, Validation, Writing – original draft. LB: Resources, Writing – original draft, Validation. YT: Validation, Writing – review & editing. MQ: Supervision, Writing – review & editing.
